# “Strangers in the ER”: Quality indicators and third party interference in Dutch emergency care

**DOI:** 10.1111/jep.12900

**Published:** 2018-03-06

**Authors:** Floortje B. Moes, Eddy S. Houwaart, Diana M. J. Delnoij, Klasien Horstman

**Affiliations:** ^1^ Research School CAPHRI, Department of Health, Ethics, and Society Maastricht University Maastricht The Netherlands; ^2^ Tranzo (Scientific Centre for Care and Welfare) Tilburg University Tilburg The Netherlands; ^3^ National Health Care Institute Diemen The Netherlands

**Keywords:** emergency care, evidence‐based medicine, health insurance, health services research, quality indicators

## Abstract

**Rationale, aims, and objectives:**

This paper examines a remarkable dispute between Dutch insurers, hospitals, doctors, and patients about a set of quality indicators. In 2013, private insurers planned to drastically reform Dutch emergency care using quality indicators they had formulated drawing from clinical guidelines, RCTs, and systematic reviews. Insurers' plans caused much debate in the field of emergency care. As quality indicators have come to play a more central role in health care governance, the questions what constitutes good evidence for them, how they ought to be used, and who controls them have become politically and morally charged. This paper is a case study of how a Dutch public knowledge institution, the National Health Care Institute, intervened in this dispute and how they addressed these questions.

**Method:**

We conducted ethnographic research into the knowledge work of the National Health Care Institute. Research entailed document analysis, participant observation, in‐depth conversations, and formal interviews with 5 key‐informants.

**Results:**

The National Health Care Institute problematized not only the evidence supporting insurers' indicators, but also—and especially—the scope, purpose, and use of the indicators. Our analysis shows the institute's struggle to reconcile the technical rationality of quality indicators with their social and political implications in practice. The institute deconstructed quality indicators as national standards and, instead, promoted the use of indicators in dialogue with stakeholders and their local and contextual knowledge.

**Conclusions:**

Even if quality indicators are based on scientific evidence, they are not axiomatically good or useful. Both proponents and critics of Evidence‐based Medicine always feared uncritical use of evidence by third parties. For non‐medical parties who have no access to primary care processes, the type of standardized knowledge professed by Evidence‐based Medicine provides the easiest way to gain insights into “what works” in clinical practice. This case study reminds us that using standardized knowledge for the management of health care quality requires the involvement of stakeholders for the development and implementation of indicators, and for the interpretation of their results.

## 1 INTRODUCTION

In response to growing demands to achieve cost control, safety, and transparency, quality indicators (or “performance measures”) have become increasingly important in the governance of health care. Quality indicators provide a means for care providers, decision makers, and purchasers to measure, compare, and improve the quality of care.^1^, ^2^, ^3^ Experts agree, both in Dutch context,^4^, ^5^ and internationally,^6^, ^7^, ^8^ that indicators are ideally based on a clinical guideline, or—in absence of a guideline—on the best available scientific evidence with regard to quality of care. Although quality indicators are not directly linked to the clinical literacy movement that Evidence‐based Medicine (EBM) originally set out to be,^9^, ^10^ some have called indicators a “branch” of EBM^11^ as they follow the same logic: clinical science can determine “what works” and parameters based on these scientific findings can form an objective standard for provider behavior.^12^, ^13^ Quality indicators developed according to an “evidence‐based approach”^6^, ^7^ are generally reputed as a technical measuring device to evaluate the quality of care providers.

As quality indicators have come to play a more central role in health care governance, the questions what constitutes good evidence for these parameters, how indicators ought (and ought not) to be used, and who controls them have become politically and morally charged. In the Netherlands, these questions were brought to a head when, in 2013, private insurers planned to drastically reform the sector using a set of quality indicators. The Association of Dutch Health Insurers had formulated these indicators drawing from clinical guidelines, RCTs, and systematic reviews from the field of emergency care. Insurers used the indicators to negotiate which hospitals would preferably provide emergency services for multi‐trauma, acute myocardial infarction, cerebrovascular accident, (ruptured) abdominal aortic aneurysm, natal care, or hip fracture. The indicators substantiated insurers' argument that the centralization of complex emergency care in few specialized hospitals would lead to better and cheaper care.

Dutch doctors and professional organizations working in the field of emergency care strongly contested the accuracy and appropriateness of the indicators and insurers' use of them. “That [plan] is unacceptable and must disappear from the agenda… not because we have anything against health insurers, but because there is not a shred of evidence that this plan provides patients with better quality or accessibility,” a spokesperson for hospitals in the Northern region claimed.^14^ Others called insurers' plans “absolutely pretentious.”^15^ Hospitals and medical specialists were concerned about insurers' lack of medical‐technical and practical insights into emergency care and feared for the quality of (and access to) emergency services.^16^, ^17^

To solve the debate between insurers, hospitals, doctors, and patients about the indicators, a public knowledge institution, the National Health Care Institute (Zorginstituut Nederland), intervened. This public knowledge institution is, amongst other things, responsible for the organization of understandable, comparable, and unambiguous information about the quality of care. A special committee of medical specialists was installed at the institute to assess the quality indicators.

In this paper, drawing from ongoing ethnographic research into the work of the National Health Care Institute, we analyse the committee's assessment of said indicators and how they addressed the questions of what constitutes good evidence for quality indicators, how indicators ought to be used, and who controls them. We conceptualize the work of the committee not as a technical exercise, but as socio‐political work. In the next section we, first, explain our theoretical background and methodology. Then, we introduce the debate about the quality indicators in more detail and explain the role of the National Health Care Institute in the Dutch health care system. After that, we analyse how the committee assessed the quality indicators and problematized insurers' strategy to use quality indicators to centralize emergency care in the Netherlands.

## 2 THEORY AND METHOD

Drawing on insights from the field of Science and Technology Studies (STS), we conceptualize the controversy about quality indicators as a socio‐political event. STS is an interdisciplinary field that examines the transformative power of science and technology to (re)arrange contemporary societies.^18^ Evidence is often invoked as a demarcation between objective knowledge and normative assumptions. Yet, quality indicators are developed at the very junction between fact and value. Therefore, in STS, standards like quality indicators are taken as socio‐political tools and not as merely technical measuring devices.^19^ Indicators are socio‐political entities, because they have financial, social, political, and moral consequences and “restructure the environments of which they become a part.”^20^ For example, when health care managers use mortality rates of myocardial infarction as an indicator to measure the quality of hospitals, this can have far‐reaching consequences for those working in those medical centers: it can redirect patient flows, reshuffle the social hierarchy between doctors, change funding opportunities and a hospital's legitimacy to work within the field of cardiology. As quality indicators “define worth” and structure the world accordingly, they inevitably become “a site of tensions, risk and uncertainty.”^21^ Taking this perspective, we study the assessment of quality indicators not as a technical exercise, but as socio‐political knowledge work and a process of negotiating societal values.

While knowledge institutions are generally tasked to give technical advice, their work in the science‐policy nexus inherently entails the balancing of scientific and public values.^22^ This paper is based on ongoing ethnographic research into these socio‐political knowledge work of the National Health Care Institute, a public knowledge institution in the Netherlands.^23^ From October 2013 to September 2017, management of the institute provided the first author with an in‐house desk (for 1‐3 days a week), a digital workplace and access to archives to do intensive fieldwork within the institute. The first author attended public and closed meetings of internal working groups (weekly), the executive board (2‐weekly), as well as expert meetings of the institute's advising committees (Package Advisory Committee, Quality Council, Scientific Advisory Committee, Health Care Professions Committee), staff fora, and informal lunches. As the controversy about quality indicators for emergency care was a flagship case for the National Health Care Institute, we decided to focus part of the study on this case.

In 3 consecutive stages, the first author collected empirical data between October 2013 and August 2016 on the debate about insurers' quality indicators and the institute's engagement with it through in‐depth conversations, document analysis, participant observation, and formal interviews. First of all, to get familiar with the case, the fist author had informal conversations with involved staff members and directors at the institute. She delved into the institute's archives to retrieve all relevant written material referring to the case, including internal and external emails, minutes of meetings, official, and internal documents. The author also searched for public reports and articles in newspapers and professional journals that reported about insurers' indicators for emergency care and the institute's work in this regard. This resulted in a dossier of over 450 pages. Secondly, to gain more direct experience with the institute's involvement in the debate, the first author attended an executive board meeting on the issue, plus 4 meetings of the Quality Council in which insurers' indicators were discussed. She also attended 2 days of consensus meetings that the institute organized with field parties to discuss quality standards for emergency care. Thirdly, in order to triangulate our observations and documentary research, we did formal, semi‐structured interviews. Through “purposive” sampling^24^ of information‐rich informants, we selected 5 key persons: 2 members of the Quality Council, 2 staff members, and the council's scientific secretary.

The first, second, and fourth author engaged in an iterative process of joint close reading of field notes, reports, policy documents, minutes, and interview transcripts. (The third author engaged in discussions of the final drafts.) The leading questions in the analysis were how the institute engaged with the debate about quality indicators, what the committee of medical specialists decided about the accuracy and appropriateness of these indicators for the (re)organization of Dutch emergency services, and why they did so. We aimed to understand how, in the Netherlands, a public knowledge institution addressed the simultaneously technical and socio‐political questions of what constitutes good evidence for quality indicators, how indicators ought to be used, and who controls them. Ultimately, as a “member‐check,” we sent a written version of the analysis to involve staff members and interviewees to test our analysis with them (face‐to‐face/email/ telephone).

## 3 RESULTS

### 3.1 The ER controversy in the Netherlands

Private insurance companies play a prominent role in the Dutch health care system of managed competition, since the introduction of the 2006 Health Insurance Act (Zorgverzekeringswet). A system of universal mandatory health insurance obliges all citizens to take out basic health insurance provided by private insurers. Competition was introduced on 2 levels. Citizens have to choose between competing health insurance companies during a yearly open enrollment period, and insurers are expected to negotiate price, service, and quality of care with providers on behalf of their insured clients.^25^ Despite heavy regulation, insurers and hospitals are free to negotiate prices and selectively contract a range of hospital care products. “Selective contracting” is a vital aspect of the Health Insurance Act. Insurers can steer their customers away from hospitals that do not reduce their prices or improve quality. This way, selective contracting is thought to stimulate both quality and efficiency.^26^

It was in their role as selective contractors that in 2013, the Association of Dutch Health Insurers (Zorgverzekeraars Nederland, hereafter “ZN”) published its “*Quality Vision for Emergency Care*,”^27^ deriving and formulating quality indicators from the scientific literature, to reorganize emergency services along efficiency lines. According to ZN, the centralization of complex emergency care in few specialized hospitals would lead to better and cheaper care. In its report, ZN focused on urgent neurological, cardiologic and vascular surgical care, traumatology, and obstetrics. (Emergency paediatrics and emergency psychiatry were not addressed in ZN's plans, because paediatric care in the Netherlands is already centralized, and psychiatric care has its own specific organizational structure.^27^) The report contained 6 specific sets of quality indicators: for measuring the quality of care for multi‐trauma and hip fracture (traumatology), acute myocardial infarction (emergency cardiology), cerebrovascular accident (emergency neurology), (ruptured) abdominal aortic aneurysm (vascular surgical care), and natal care (obstetrics). For example, for multi‐trauma, the insurers specified that good care required the following: a trauma center with a treatment team, 24‐hour accessibility of an internal trauma team; the presence in hospital of the required specialists within 15 minutes; mobile medical team; adequate facilities (like a CT‐scan in the emergency room; an operating theatre and intensive care unit next to emergency room). Quality of care would be measured based on 30‐day mortality after (multi‐)trauma. In order to guarantee quality, a hospital was required to treat 240 to 480 multi‐trauma patients per center, per year.^27^

ZN substantiated the indicators by referring to Dutch, European, and international guidelines and a vast number of scientific publications, including RCTs, systematic reviews, and cohort studies. According to ZN, private insurers could use these indicators for selective contracting of emergency cardiology, emergency neurology, traumatology, urgent vascular surgical care, and obstetrics. They also proposed to centralize these services in specialized centers if the proposed quality indicators indicated that this was desirable.^27^ By the end of 2013, insurers started negotiating (using said indicators), which hospitals would maintain a fully equipped emergency unit, and which hospitals would lose part of their emergency care.

A journalist reported: “In Rotterdam most complex emergency care will move to Erasmus Medical Center. Soon four times as many people will be going there with a stroke. Five hospitals will lose stroke care.”^28^

A hospital director stated: “Emergency room closed, no more obstetrics, nor stroke care, no balloon angioplasty, and even broken hip operations will have to be done elsewhere”.^15^

The plans caused much debate in the field of emergency care. From the exchange of arguments in professional media, it became clear that the accuracy and use of ZN's indicators was contested by Dutch professional organizations. The Dutch Hospital Association (NVZ) emphasized that such indicators need to be well‐founded and was concerned about insurers' lack of medical‐technical and practical insights into emergency care.^16^, ^17^ NVZ criticized that insurers gave little credence to the fact that it is often difficult to predict a patient's diagnosis before he/she enters a hospital. A patient with acute abdominal pain could suffer from a wide variety of conditions ranging from gastroenteritis to abdominal aortic aneurysm. The director of the NVZ rhetorically stated: “will concentration mean, for instance, in respect of abdominal aneurysms, that all patients with stomach pain must be sent immediately to hospitals with an emergency room for complex acute care?”^15^ In another line of critique, the Dutch Order of Medical Specialists (OMS) claimed that a hospital that loses its contract for the treatment of, for example, myocardial infarction would have to deal with a “cascading effect,” meaning that the general cardiology in that hospital could deteriorate or vanish completely from that hospital, too. The OMS urged that “a hospital is a careful construction of building bricks, removing just one brick can cause everything to collapse.”^29^ Of course, apart from quality concerns, hospitals and specialists had their own interests at stake in the debate.

In 2014, Dutch professional organizations, the NVZ and OMS, appealed to the Authority for Consumers and Markets (ACM) to express their concerns about insurers' plans. ACM is the regulatory institute that oversees markets, fair competition and consumer rights. The regulatory body was concerned that insurers' plans to concentrate emergency care could “reduce the choices open to patients and insured clients” and warned insurers that “implementing the proposed plans could contravene the Competition Law.”^30^ ACM decided that curtailment of consumer choice was only acceptable if it would lead to a considerable quality gain for patients. The regulatory body ordered insurers to demonstrate that this was indeed the case by providing “independent and well‐founded quality standards for emergency care”^30^ or to organize broad support for the indicators in the field. As ZN required quality indicators for their practice of selective contracting, it turned to the National Health Care Institute to organize broadly supported quality indicators.

### 3.2 The role of the National Health Care Institute

The National Health Care Institute is, amongst other things (such as the management of the Dutch basic benefits package),^23^ lawfully tasked to organize understandable, comparable, and trustworthy information about the quality of care. Since the introduction of the 2006 Health Insurance Act, reliable quality information has become a crucial ingredient for the proper functioning of the Dutch system of managed competition.^31^ Quality information provides both consumers and insurers with comparable information about the performance of health care providers in order to negotiate price and quality.^32^ In the years that followed the introduction of the Health Insurance Act, the health care sector developed all kinds of quality information. The multiplicity of indicators, however, actually blocked oversight and hindered informed consumer choice.^33^ In response to this problem, the Minister of Health proposed to centralize the organization of quality information, and on April 1^st^ 2014, the National Health Care Institute was established. The institute was lawfully tasked to organize understandable, comparable, and trustworthy information about the quality of care, and has what is called “overriding authority”: it can authoritatively lay down indicators when field parties fail to deliver them or to reach consensus. If the institute uses this “overriding authority,” the development of quality standards or indicators is put in the hands of the Quality Council, an advisory committee of the National Health Care Institute.

It was from their role as coordinator of quality information that the institute, was asked to assess the quality indicators for emergency care. After several rounds of meetings and consultations facilitated by the institute, the professional organizations of insurers (ZN), patients (NPCF), medical specialists (OMS), hospitals (NVZ), and academic hospitals (NFU) failed to reach agreement on insurers' quality indicators for emergency care. That is to say, the specialists, (academic) hospitals and patient organizations submitted their own revised set of indicators, but without the support of the insurers. As a result, the institute used its “overriding authority” and asked the Quality Council to review the revised indicator set. The council, in turn, installed a group of 13 medical specialists to do the formal assessment. To safeguard representativeness, the council selected specialists from different relevant medical fields in emergency care and from both peripheral and academic hospitals. Also, the selected specialists could not be formally affiliated to any of the stakeholders that proposed the indicators. Next, we show how the committee assessed the quality indicators and problematized insurer's strategy to use them to centralize emergency services in the Netherlands.

### 3.3 Problem 1: Scope of indicators

The committee installed by the Quality Council was tasked to assess whether the indicators were “evidence‐based” and adequate for measuring the quality of emergency care. From our interviews, observations, and documentary research, it became clear that the committee actually found that most of the indicators were, as staff commented, “not unreasonable,” as there was sufficient evidence supporting the indicators. The accuracy of the individual indicators was not the primary problem according to the committee. They did have a problem with the *scope* of the indicators. Next, we illustrate this with an example.

Insurers proposed to measure, for example, the quality of care for acute myocardial infarction (AMI) by looking at how a hospital scored at the following:

- *availability of a Cardiovascular Intervention Center, Emergency Cardiovascular Care (ECC), Cardiac Care Unit (CCU), and a cardiac rehabilitation ward (structural indicators);*
- *percentage of ST‐Elevation Myocardial Infarction (STEMI) treated with primary percutaneous coronary intervention (PCI), and hospital's percentage of medication after hospital discharge for AMI (process indicators);*
- *AMI mortality rate after 30 days, patient‐reported functional health after AMI, and percentage of PCI re‐interventions (outcome indicators);*
- *hospital's/ cardiologist's annual number of PCI‐treatments performed (600 PCIs per hospital /150 PCIs per cardiologist) (volume norms)*.^27^

Our interviewees explained that, while these indicators make sense in themselves, the quality of the treatment of myocardial infarction depends on the entire trajectory from incident to aftercare, not just on what happens inside a hospital. Staff members said that insurers' indicators “started in the hospital,” while “an emergency pathway starts with a demand for emergency care, so it starts at home perhaps, or in the street… or under a car …”. A committee member explained in an interview that the quality of treatment for myocardial infarction,

“starts with a patient raising the alarm in good time, after which the GP takes a look, who then refers the patient to hospital in good time, and all of that finally determines the outcome of a myocardial infarction. If you judge a hospital based on, for instance, survival rates for myocardial infarction, well then you would also have to take into account the part of the chain before the hospital… if people call in the GP too late, or the GP doesn't do his job properly, you will be running the risk that indicators will work against hospitals. Well, you have to realize this, if you are going to make use of this sort of indicator.”

The committee therefore broadened the scope of the indicators. In their final report “*Excellency Demanded for Emergency Care*,”^34^ the committee systematically supplemented indicators focusing on hospital performance with indicators regarding the organizational chain of emergency services (including, for example, regional ambulance services, triage systems, and urgent transferal). For AMI, for example, the committee included “written agreements about cooperation with partners in the organizational chain” of care for AMI.^34^ By doing so, the committee emphasized the importance of the network in which an emergency patient makes a journey. It is within this network that diagnoses are made and the quality of treatments takes shape. By adding “chain agreements” to the set of quality indicators, the committee required that the quality of hospitals was not only measured by hospitals' “in‐door” performance, but also measured by looking at hospitals' abilities to organize and maintain a well‐functioning network in the region.

### 3.4 Problem 2: Purpose of indicators

As we stated before, the committee found that most quality indicators were supported by evidence. This was, however, not the case for the so‐called “optimum” volume norms proposed by insurers. The committee problematized not only the lack of evidence for insurers' volume norms, but also the purpose behind the norms. Next, we elaborate on the committee's problems with insurers' “optimum” volume norms, and why they proposed “minimum” volume norms instead.

The idea behind volume norms is that hospitals treating a bigger volume of patients get better treatment results, thus, leading to better quality and efficiency. ZN wrote in their report that

“large volumes will make it possible to use an expensive infrastructure […] more effectively. At the same time, a larger volume will also deploy personnel (specialists and specialist nurses) that is scarce (and becoming even scarcer) more effectively.”^27^

ZN proposed “optimum” volume norms describing at what number of patients the quality and efficiency of emergency services would be optimized. ZN recommended for stroke, for example, that a hospitals would have to treat at least 350 patients with cerebrovascular accident (CVA) per year.^27^ For (ruptured) abdominal aortic aneurysm (rAAA), insurers claimed that hospitals would have to treat at least 15 ruptured abdominal aortic aneurysms and a total of 33 abdominal aortic aneurysms or more to deliver good quality care for these patients.^27^According to ZN, their “optimum” volume norms were “an estimate of what is a sensible level of emergency care concentration in order to book both quality and efficiency gains.”^27^

Insurers' volume norms, however, were strongly contested by field parties. According to staff members the “biggest stumbling blocks really were the volume criteria” in the debate about quality indicators. The committee's secretary recalled that “the volume norms… that was what led to discussions,” because these norms had far‐reaching consequences. The chairperson to the committee explained in an interview that if insurers would have selectively contracted emergency care on the basis of these optimum volume norms, this “would have resulted in a lot of casualty departments shutting down … it would have been a ravage … lots of them would have closed because they were simply unable to fulfil the criteria.”

The committee installed by the Quality Council was tasked to assess whether the volume norms were “evidence‐based” and adequate for measuring the quality of emergency care. According to the committee's secretary there was “insufficient evidence” supporting these optimum norms. It became clear from our interviews, observational, and documentary research that the committee did not only have a problem with the lack of evidence for insurers' volume norms, but also with the *purpose* behind these “optimum” norms. A committee member explained that “based on a principle of solidarity or collectivity, you might actually think that [care] should be ‘good enough’ everywhere,” and not necessarily “optimal.”

In their final report, the committee proposed “minimum” volume norms following the advice of the scientific associations of the different fields of medical expertise. Staff members explained that scientific associations “often stipulate minimum norms” and that “these are often so low that everyone fulfils them.” The purpose of minimum norms is not to optimize quality and efficiency, but to secure a minimum level of safe and reliable emergency services. Working from this idea, the committee lowered insurers' optimal volume norms to minimum norms. For CVA, for example, the committee followed the Dutch Association for Neurology (NVN) that advised a minimum volume of 100 patients with cerebrovascular accident (CVA) per year per hospital. At the same time, the committee raised the minimum volume norm for (ruptured) abdominal aortic aneurysm (RAAA). Working from that same idea that volume norms serve the purpose of securing safe and reliable emergency services, the chairperson explained, the committee thought: “with aneurysm for example… this is a life threatening procedure… and if you do less than 20 of those in a hospital [per year]…. that is just unwise.” By stipulating minimum volume norms, the chairperson to the committee claimed that on the one hand: we “made sure no damage was inflicted by units having to close down or being no longer able to provide care, units that I felt – we all felt – were capable of providing good care…” on the other hand: “our norms prevented aneurysm operations from still being carried out in [city X], while they really should be taken to [city Y], because then the survival of these people is actually much higher.”

Apart from the lack of evidence supporting the optimum volume norms of ZN, the committee problematized the purpose behind optimum norms. Instead, they stipulated minimum norms, following the scientific associations of the respective field of medical expertise. While the purpose of insurers' optimum volume norms was to organize optimal quality and efficiency in emergency care and thus optimize welfare economics, the purpose of the minimum volume norms endorsed by the committee echoed the rationale of another public responsibility: a collective duty to secure access and availability of “good‐enough” emergency services for all.

### 3.5 Problem 3: Use of indicators

Finally, our research showed that the committee problematized insurers' unilateral use of the quality indicators. In their final report, the committee revised and laid down quality indicators for emergency care and stressed the importance of collectively weighing the applicability of the indicators and their fit with regional context.^34^

ZN designed quality indicators to enable “insurers to shape their selective care purchasing.”^27^ On the basis of these indicators, insurers had started to compare hospitals in the different regions and started negotiating selective contracts. Field parties, however, considered insurers' initiative a top‐down exercise. A hospital director recalled the negotiations with insurers: “there were lists with green and red ticks” and objected: “you can't throw down some sort of blueprint from your ivory tower.”^14^

According to the chairperson to the committee, the problem was that insurers were acting unilaterally using standardized knowledge, while they were actually at a distance from the primary care process and had no hands‐on experience of emergency care:

“This was just one party… and a party that was at a considerable distance… and because [the insurers] are at such distance, their only weapon is population data from the evidence‐based medicine world… which is standardized… if someone has no hands‐on experience and has never actually been to an emergency room… then these are the only available data for him to use. But, then he forgets the knowledge sources that are somewhat harder to exploit for him, but that are very important too…’.

With “evidence‐based medicine as the foundation of the quality movement,” the chairperson claimed, focus has moved to “standardized protocol quality.” But in applying such standards “local data are important” and collaboration in the region is necessary to exploit sources of local, tacit, and contextual knowledge. In their final report, the committee stressed that the quality indicators they proposed were to be used by professionals to discuss collectively how best to organize emergency services in the particular regions.^34^ Rather than a unilateral tool for selective contracting, the committee proposed the indicators as a tool for dialogue and learning, allowing regional parties to gain insight into opportunities to improve situational quality.

## 4 DISCUSSION

The National Health Care Institute is a public knowledge institute that is, amongst other things, lawfully tasked to organize trustworthy information about the quality of care. We studied how this body intervened in a debate about the quality of emergency care, and in that context we analysed their assessment of quality indicators as socio‐political knowledge work. Our analysis of their work showed a struggle to reconcile the technical rationality of quality indicators with their social and political implications in practice. The committee installed by the National Health Care Institute problematized not only the evidence behind some of the quality indicators, but also and especially their *scope*, *purpose* and *use*. According to the chairperson, the committee “put the evidence into a bit of perspective.”

In scientific literature on the development of quality indicators it has been recognized (albeit in different wording) that indicators operate at the very junction between fact and value.^3^, ^6^, ^7^, ^35^ Although this paper describes a single case, it serves as a real‐life example of what experts on indicator development have long recognized: that even if quality indicators are based on solid scientific evidence, they are not “axiomatically good”^36^ or useful. Indicators developed for 1 purpose (eg, measuring the quality of care for myocardial infarction or stroke) may be inappropriate for a different application (eg, measuring the ideal level of concentration of these emergency services).^7^ Furthermore, indicators can serve a wide array of values; not all of them equally desirable from the perspective of public health services. The proper use of quality indicators in a public context requires the involvement of stakeholders in the development of indicators, the collective formulation of objectives, and the use of local, tacit, and contextual knowledge to aid both the implementation of indicators and the interpretation of their outcome.^36^

The socio‐political work of the National Health Care Institute reflects specific characteristics of Dutch health care. The Netherlands has a system of managed competition in which insurers are expected to stimulate quality and efficiency in health care through, for example, selective contracting. Private insurers, however, still lack public trust in the Netherlands.^37^ The Dutch healthcare system is highly regulated and private insurers operate under strong legislation and are critically watched by all kinds of professional organizations and state institutions. The Dutch have, furthermore, a culture of consensual policy‐making called “polderen.”^38^ Not every health care system is as much disposed to inclusive dialogue. The attempt by insurers to corroborate concentration plans with quality indicators reminds us that—with the marketization of healthcare—quality indicators can easily align to match the logic of the market. This case study shows that it requires specific knowledge work and dialogue to realign such scientific tools to make them socially robust and serve a public purpose.

### 4.1 Concluding remarks

Quality indicators are not directly linked to EBM. They do, however, follow the same logic: clinical science—ideally statistical population research—can determine “what works” and parameters based on these scientific findings can form an objective standard for provider behavior. Both proponents^9^, ^10^, ^39^ and critics^40^, ^41^ of EBM have long problematized the uncritical use of statistical population research and the evaluation of medical practices based on probabilistic knowledge.^12^, ^13^ However, for non‐medical parties who have no access to the primary care processes, this type of knowledge provides the easiest way to gain insights into “what works” in clinical practice. EBM's emphasis on standardized, impersonal research procedures suggests the possibility of separating “expertise from expert and knowledge from knower” and that it is possible to distill “medical truth outside the clinical encounter.”^13^ As a result of this uncoupling of expert and expertise, the use of statistical population research has given outsiders to health care the scientific authority to act independently in a field where—originally—they had none.^42^ While outside interference into clinical practice is of all times,^43^ critics^40^, ^41^ feared that EBM's standardization of medical knowledge, would allow “strangers”—like inspectorates, policymakers, or insurers—to “regulate the field of healthcare and hold it accountable using evidence‐based parameters formulated by the professions” themselves.^20^ This case study reminds us that the interplay of forces between the uncritical use of standardized medical knowledge, the growing use of quality indicators, and the marketization of health care continues to require our explicit attention.
